# The social gradient in doctor-patient communication

**DOI:** 10.1186/1475-9276-11-12

**Published:** 2012-03-12

**Authors:** Evelyn Verlinde, Nele De Laender, Stéphanie De Maesschalck, Myriam Deveugele, Sara Willems

**Affiliations:** 1 Department of Family Medicine and Primary Health Care, Ghent University, Ghent, Belgium; 2 Verlinde Evelyn, Department of Family Medicine and Primary Health Care, Ghent University, UZ-1 K3, De Pintelaan 185, B-9000 Ghent, Belgium

**Keywords:** Communication, Physician-patient relations, Social class

## Abstract

**Objective:**

In recent years, the importance of social differences in the physician-patient relationship has frequently been the subject of research. A 2002 review synthesised the evidence on this topic. Considering the increasing importance of social inequalities in health care, an actualization of this review seemed appropriate.

**Methods:**

A systematic search of literature published between 1965 and 2011 on the social gradient in doctor-patient communication. In this review social class was determined by patient's income, education or occupation.

**Results:**

Twenty original research papers and meta-analyses were included. Social differences in doctor-patient communication were described according to the following classification: verbal behaviour including instrumental and affective behaviour, non-verbal behaviour and patient-centred behaviour.

**Conclusion:**

This review indicates that the literature on the social gradient in doctor-patient communication that was published in the last decade, addresses new issues and themes. Firstly, most of the found studies emphasize the importance of the reciprocity of communication.

Secondly, there seems to be a growing interest in patient's perception of doctor-patient communication.

**Practice implications:**

By increasing the doctors' awareness of the communicative differences and by empowering patients to express concerns and preferences, a more effective communication could be established.

## Introduction

In 1977 a commission under the lead of Sir Francis Black published the famous Black report, illustrating the existence of a social gradient in health in the UK. The publication of this report was the start of a new wave of research on social inequity [1]. Since then many studies have confirmed the gradient in health between social classes [2-4]. Health differences between social groups due to underlying social mechanisms such as differential access to care, social exclusion or poverty are a matter of major concern in today's public health research but in spite of marked health improvements of the overall population and efforts to overcome health inequalities, higher morbidity and mortality rates for the socio-economically disadvantaged are still found [5-8]. The causes for these inequalities in health are multiple and complex: a different distribution of power and resources among social classes, different levels of exposure to health hazards, same level of exposure leading to differential impacts, life-course effects and different social and economic effects of being sick [2,9-16]. A prerequisite for equity in health is equity in health care, defined as equal care for people in equal need of care. As Dahlgren and Whitehead quoted: "Equity in health care includes fair arrangements that allow equal geographic, economic and cultural access to available services for all in equal need of care" [17].

An essential component of the delivery of health care is the relationship between the patient and the health care provider [18]. Several studies on communication in health care have repeatedly shown the importance of the doctor's communication skills [19]. By communicating with a patient, a physician gets to know the patient's problem and creates a therapeutic relationship necessary for its management and, if possible, its solution [20]. The quality of the relationship between a doctor and a patient is a key factor in the effectiveness of care. Good doctor-patient communication is associated with a higher level of patient satisfaction and better compliance [19,21]. Furthermore, optimizing doctor-patient communication can lead to better patient health and outcomes [22,23].

Available evidence suggests that low-income populations and people without health insurance report lower communication satisfaction and a reduced access to care [24]. In recent years the importance of social inequalities in the physician-patient relationship has frequently been the subject of research [19,21,24]. A previous review showed that doctor-patient communication indeed varies according to the social class of the patient [25].

Considering the increasing importance of social inequities in health care, an actualization of the review seemed appropriate.

If differences in the physicians' communicative behaviour vary according to the socio-economic status of the patient, this could be a new focus in the battle against socio-economic inequities in health.

In this paper we want to answer the following questions based on a systematically review of the literature:

Does the doctor-patient communication varies according to the socio-economic status of the patient?

If so, which aspects of the consultation are affected?

Are the findings of studies published after 2002 different than those of the publication of the first (and the latest) review on this topic [25].

## Methods

Before starting the review, a protocol was developed, including the following steps.

### Search strategy

In step one, a systematic search in MEDLINE, PsycINFO and Web Of Science was conducted to identify publications on doctor-patient communication and social class of the patient. The following search strings were used:

MeSH: communication AND (physician-patient relations OR provider-patient relations OR physician-family relations) AND (social class OR socio-economic factors)

Text-words: (doctor-patient communication OR physician-patient communication OR provider-patient communication) AND (social class OR socio-economic status).

The search was limited to publications from 1965 on. No specific search software was used.

Articles that were not original research articles, opinion articles and reviews were excluded. Furthermore, the search strategy was narrowed to studies performed within industrialized countries.

### Outcome measures

To make the comparison of results possible, articles were included when they mentioned the interaction between the socioeconomic status (SES) of the patient or one of its indicators (educational level, income or occupation) as well as determinants of doctor-patient communication. At the initial stage, doctor-patient communication was not yet defined into specific categories, in order to obtain a wide range of studies. This means that studies mentioning any form of doctor-patient communication were selected. We included as well studies from primary care as specialist care to gain insight in the overall social gradient in doctor-patient communication. This resulted in a list of 129 articles.

### Study selection

**Figure 1:**
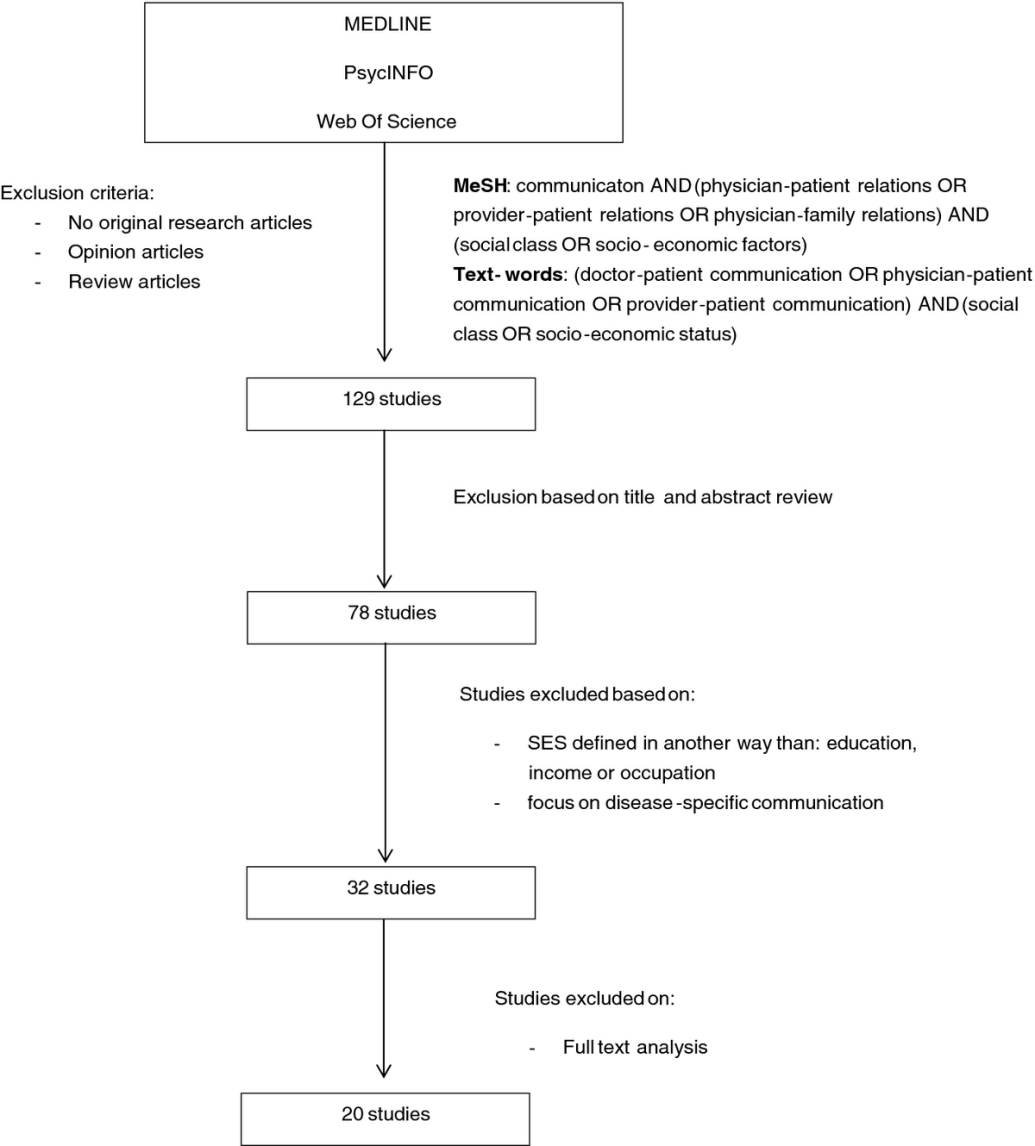
**Selection procedure**.

Figure 1 provides an overview of the study selection process. Of the 129 studies under review, 51 were excluded based on title and abstract review since they were after all not related to doctor-patient communication and social class. The abstracts of the remaining 78 publications were screened for explicit references to social class related concepts (education, income or occupation) and doctor-patient communication. Fourty-six articles determining SES by other variables than education, income or occupation (e.g. race, gender, health literacy) and articles focussing on disease-specific communication were excluded. In the last step of the selection process, an independent full text analysis of the remaining 32 publications was performed independently by two of the researchers to confirm the relationship between social class and doctor-patient communication in the publications. Publications labelled as "doubtful relevance concerning social class and doctor-patient communication" by one of the reviewers, were discussed until consensus was reached. Twelve publications were rejected in this phase. Eventually, 20 publications were labelled as relevant to asses doctor-patient communication and social class of the patient (Table 1).

**Table 1:** Overview of the selected articles

First author (Ref. nb.)	Setting	Method	Nb of patients	Variable SES	Variable communication doctor	Variable communication patient	Information on validity and reliability	Conclusion of the study
Hall	professional health care providers	meta-analysis	157 (mean)	social class indices, education or income	information giving, question asking, task and interpersonal competence, partnership building and socio-emotional behaviour		Correlation and standard norm deviate was extracted for each study if possible	Higher social class: more overall communication and more information.

Street	primary care	audiovisual analysis	41	education	information giving (diagnostic, treatment, procedural)	communicative style: affective expressiveness	Unitizing reliability for utterances: Cohen's kappa = 0.84 Reliability categorizing:	Higher educated patients: more diagnostic and health information.
							Physician information giving: 0.82 Partnership building: 0.87 Patient's opinion giving:0.82	More question asking by patient leads to more information giving.
							Patient affective expressiveness: 0.75 Patient's question asking: 0.96	No relation between educational level and question asking

Street	multipurpose clinic, pediatric consultation	audiotapes	115	educational level	partnership building	parent's question asking and opinion giving	Reliability: - physician response (0.72-0.95) - patient response (0.68-0.91)	Higher education: more expressive, higher level of opinionated and asking more questions.
								Personal characteristics less influence on physician response than own communication behavior?

Martin	primary care	questionnaires	1972	occupation	listening, explaining, advice giving, examination	listening, explaining, advice giving, examination m	No information available	Patient perception of consulation: emphasis on prescribing, reassuring and referring
								Physician's perception of consultation: emphasis on active listening, supporting and giving advice.
								Higher social class: more examination, listening and explaining.
								Patients perceive no difference

Fiscella	primary care	direct observation, chart audits, patiënt reports	2538	education	time use, preventive tasks, satisfaction, attributes of primary care	Interpersonal communication, patient satifaction	Time use: Davis Observation Code Attributes of Primary care: Components of Primary Care Instrument Patient satisfaction: items from the Medical Outcomes Survey	Lower education more physical examination and nutritional counseling, less time on questions, assessing health knowledge, negotiation and counseling, chatting and screening tests.
							Reliability: doctor satisfaction = 0.90, nurse satisfaction 0.72	Same satisfaction as higher educated.

Taira	Employees	questionnaires	6549	income	discussion of health risk	health risk behaviours	No information available	High income: more diet and exercise discussion.
								Lower income: more smoking discussion

Pendleton	primary care	videotaped consultations	79	social class	amount of information given to the patiënt		No information available	High SES: more explanations

Street	primary care	videotaped consultations	41	education	nonverbal behaviour consistency and adaptations		Cohen's kappa: 0.82 for speaking turns and response latencies 0.71 for interruptive speakovers 0.92 for physicians' taks touch 0.85 for illustrators 0.71 for adaptors 0.79 for procimity 0.93 for body orientation 0.90 for turn duration 0.81 for response latency 0.83 for pausing within speaking 0.75 for patient's anxiety	Physicians talking with higher educated patients used more body orientated talk then they did with lower educated.

Kaplan	solo & multispecialty practices	questionnaires	8316	education	PDM (Participatory Decision-Making) style: involve them in treatment decisions, give them a sense of control over medical care and ask them to take some responsibility for care		Data from the Medical Outcomes Survey (MOS) Reliability Participatory decision making style: 0.74	Lower educated patients: less mutual decision making, less sense of control and given less responsibility.

McKinstry	primary care	structured interview, video vignettes	410	social class indices	shared decision making style		No information available	Lower educated patients: lower preference for shared decision making.

Roter	primary care	audiotape RIAS; questionnaires	537	income	narrowly biomedical, expanded biomedical, biopsychosocial, psychosocial, consumerist pattern	idem	Reliability physician: 0.76 Reliability patient: 0.81	Lower SES patients prefer narrowly biomedical pattern.

Stewart	primary care	audiotapes	140	educational level	information giving; patiënt centredness		Statement made by doctor: Bales Interaction Process Analysis Communication on drugs: scheme developed by Svarstad and refined by Scherwitz and Evans	Higer education: more explanation on drug prescription
								Low education: more emotional support

Maly	breast cancer treatment program	survey, PEPPI	327	education, income	interactive information-giving	patient-perceived self- efficacy	Self-efficacy: validated Perceived Efficacy in Patient-Physician Interactions (PEPPI) questionnaire Language: Marin Acculturation Scale with reliability of 0.99	Higher education: more interactive information giving by physician and greater perceived self-efficacy.

Piette & Schillinger [39]	department of Veterans Affairs (VA),university- based and county health care system	telephone interview	752	education	Interpersonal Processes of Care (IPC) questionnaire: general clarity; explanations; elicitation of patient's preferences, emotional support		Revised scale of the Interpersonal Processes of Care (IPC) questionnaire with reliability of 0.91	Low SES: better general and diabetes-specific communication than high SES

Jensen	primary care	survey, interview	131	education, income	explain things; listen carefully to what the patient has to say; show respect; spend enough time with the patient	idem	Questions coming from the Medical Expenditures Panel Survey	Patients with high literacy skills are more critical on their physician.
								Low income perceive some areas of tension in communication with their health care provider.

Street	primary care patients, lung cancer patients, patients with systemic lupus erythematosus	audiotapes	279	education	partnership-building; encourage patient involvement; supportive talk	asking questions; assertive responses, expressions of concern or other negative emotions	Coding system developed by Street and colleagues with reliability ranging from 0.61 to 0.97 depending on the behavior and the study	Higher educated patients are more active communicators, ask more questions and are more assertive, but they do not express more concerns
								Patients are more active communicators when physicians use partnership- building.

Siminoff	oncology practices	audiotapes, RIAS	405	education, income	educating and counseling the patient concerning biomedical and psychosocial issues-ask patients for information to indicate understanding, opinion or permission-attempting to built a relationship with the patient-engagement in conversation about the patients emotional status-gathering relevant data and information	patient communicates biomedical and psychosocial information; asking questions; building relationship with the physician, engaging in discussion; expression of feelings;	Doctor-patient communication: Roter Interaction Analysis System (RIAS)	High income patients: receive more biomedical talk, emotional talk, psychosocial counseling and education, ask more question and receive less questions about their disease than low income patients.

Murray	american households	computer- assisted telephone interview	3209	education, income	giving information; decision-making style	preferred style of decision-making; experienced style of decision-making;	No information available	High SES patients prefer shared decision making
								Lowe SES patients prefer consumerism and paternalism
								High SES patients are more likely to experience the preferred style

Bao	primary care	patient and physician surveys	5978	Income, education	self-assessment of communication; performance of communication behaviours when discussing cancer screening		Questionnaire from the Communication in Medical Care (CMC) Research Program series.	Low SES patients are more likely to discuss cancer screening then high SES patients.
								Between-physicians differences by income
								Within-physicians differences by education.

Devoe [24]	primary care	secondary analysis of data from Medical Expenditure Panel Survey (MEPS) (face-to-face interview)	16 700	educational attainment, family income	listen carefully; explain things; show respect; spend enough time with the patient		Questionnaires coming from the Medical Expenditure Panel Survey (MEPS)	Poor patients: receiving less explanations in a way they understand.

### Analysis and synthesis of the study findings

A narrative review was conducted. Meta-analysis was not attempted due to heterogeneity of populations and outcome measures among included studies. Findings were compared according to investigated communication measure and according to the social class. To cluster the communication variables, several communication assessment approaches were considered [26]. Communicative behaviour can be categorised in terms of verbal and non- verbal behaviour. Verbal behaviour can be defined as 'the spoken communication'. The verbal elements of communication can be divided into instrumental or task-focused verbal behaviour (e.g. question asking, information giving, etc.) and affective or socio-emotional behaviour (counselling, positive and negative talk, etc.) reflecting the distinction between cure and care [27,28].

However, some of the determinants of communication do not fit into the above categories but are related to the concept patient-centeredness. Patient-centeredness is about seeing the patient as a person with a unique personal history and individual needs. We can identify five dimensions: (1) using the bio-psycho-social perspective, (2) approach the patient as a whole person, (3) sharing power and responsibility, (4)building a therapeutic relationship and (5) considering the physician as a person and acknowledging the influence of its personal qualities [29,30].

The communication variables in the selected articles are classified to the following categories: verbal behaviour including instrumental and affective behaviour; non verbal behaviour and patient-centred behaviour. The selected studies were grouped and analysed by the researches according the three communication variables.

## Results

### Verbal/non-verbal behaviour

#### Verbal behaviour: instrumental behaviour

Instrumental behaviour is considered as all interactions that serve the "cure" part of the consultation. It can be defined as technically based skills that are used in problem solving e.g. giving directions, giving information, asking clarification, asking questions, counselling, etc. [26,31]. Eleven studies explored the interaction between the instrumental behaviour of the physician and/or the patients, and the SES of the patient.

A meta-analysis conducted by Hall et al. explored the correlation between physicians' communicative behaviour and the patient's outcome variables. Social class was measured by income, education or other non-specified social class indices. The study revealed a positive relationship between patient's social class and information giving. Patients of a higher social class received not only more overall communication but also more information [32]. Not only patients' social class but also his/her communication style influences the doctor-patient communication. In a study by Street et al., social class was measured by educational level. Physicians' information giving was positively influenced by the patient's communicative style such as question-asking, affective expressiveness and opinion-giving. More affective expressiveness and being assertive on the patient's side- which is strongly related to his/her educational level- leads to more information giving on the doctor's side. More educated patients receive more diagnostic and health information than their lower educated counterparts. However this study did not find a relation between the frequency of the patients' question asking and his/her educational background [33].

The fact that adaptations in the physicians' responses may, besides a function of patients' personal or social characteristics per se, also are the result of the patients' communicative actions, was confirmed by a second study by Street et al. In this study they compared the degree to which parents' personal and interactive characteristics accounted for variation in doctor-parent interactions during paediatric consultations. Social class was measured as educational level. More educated parents are not only more expressive and assertive but they also ask more questions. All three of these communication aspects lead to more information and direction giving by the physician. Additionally, this study shows that the parent's personal characteristics have less influence on the physicians' responses than their own communication behaviour [34].

Besides patient's communication style, doctors' and patients' perceptions are an important aspect of the consultation and for the outcome of the consultation. In an observational study, Martin et al. looked at how both physicians and patients perceive what happens during the consultation. Social class was measured by occupation. From the patients point of view most emphasis of the consultation is put on prescribing, reassuring and referring. Whereas physicians report that emphasis is put on active listening, supporting and giving advice. Furthermore, physicians perceived they explained and listened more to patients from higher social classes and also examined them more than patients from lower social classes, but gave the latter more "other help" which was not specified. They also said to examine more and to give less advice to patients from lower social classes. However, patients did not report having experienced any of these differences [35]. The study of DeVoe et al. where social class was determined by family income and educational level, shows different results. This study suggests that patients' perceptions of communication in healthcare settings vary widely by demographics and other individual patient characteristics. The poorest patients were less likely to report that providers always explained things so that they understood. Surprisingly, different levels of education were not independently associated with any of the investigated communication measures [36]. These results are in line with the results from the study of Fiscella et al., exploring whether educational level affected the visits of family physicians. Patients with a low educational level had a slightly larger proportion of the consultation time spent on physical examination and nutritional counselling. Less time was spent on patients' questions, assessing their health knowledge, negotiating and counselling, chatting, and less screening tests were provided to them. One could say that less educated people are approached in a more directive way during the consultation. Less educated patients also saw their expectations less met during the consultation, although they were as satisfied as the more educated patients [37]. When looking at the outcome of the consultation, Maly et al. studied the impact of physician-patient communication on women's receipt of, or planning for, breast reconstructive surgery. Social class was determined by educational level and was included as a potentially confounding factor. Women who had graduated from high school were more likely to report planning of breast reconstructive surgery. This is positively associated with interactive information-giving by the physician and greater patient perceived self-efficacy. These two communication factors weakened the negative influence of education barriers. Empowering aspects of patient-physician communication and self-efficacy may overcome the negative effects of a lower education on receipt or planned breast reconstructive surgery [38].

In line with the different consultation style, Taira et al. investigated whether the patients' income level had an influence on the physicians' discussion of health risk behaviours. Concerning patients at risk, physicians tended to discuss diet and exercise more with high income patients and smoking more with low income patients [39]. Discussing health risk behaviour is very important in consultation, especially for chronic conditions like diabetes. In a cross-sectional survey by Piette et al., general communication processes and diabetes-specific communication was examined. Socio-economic status was measured by means of educational achievement. Patients with lower education levels reported better general and better diabetes-specific communication than their less-vulnerable counterparts. This could be due to the fact that these patients have lower expectations of their patient-provider relationship or greater discomfort with criticizing them. Another introduced explanation could be that health care providers spend more time counseling patients which they perceive as in need for extra attention or explanation [40].

Pendleton et al. considered four types of information giving. SES was measured by social class. There was a significant difference in voluntary explanations given to patients from different social classes, independent of the different types of problems; higher SES patients receive significantly more explanations even when the explanation was not explicitly requested by the patient [41]. Also the study from Siminoff et al. where social class was measured by income and educational level showed that more biomedical talk was provided to higher income patients compared to medium and low income patients and to patients with higher educational achievement. In general, physicians provided little psychosocial counseling and education, however, they provided more to their high and medium income patients as compared to low-income patients. Patients that had more than a high school education and patients that reported a medium or high income asked more questions and showed more proactive behavior such as volunteering information to the physician unasked. Physicians on their side asked less educated patients and low income patients more questions about their disease and medical history [42].

#### Verbal behaviour: affective behaviour

The affective behaviour in doctor-patient communication is part of the emotional domain [19] and consists of all forms of social behaviour and social talk. Possible affective expressions are: showing concern, reassurance, reflection, signs of agreement or disagreement and paraphrasing [27,28]. Only three studies investigated the effects and outcomes of affective behaviour.

The meta-analysis by Hall et al. (supra) explores the socio-emotional behaviours such as social talk and positive and negative talk. Although a link between the aspects of affective behaviour and the patients' satisfaction and compliance can be identified, none of these determinants were found to be related to any determinant of the patients' social class [32]. On the other hand, the studies of Street et al. (supra) concluded that doctors provided more comments of reassurance, support and empathy to the parents of children with cancer which were more affectively expressive (more specifically who expressed more negative affect). As patients with a higher educational level are more affectively expressive than their counterparts, it can be assumed that physicians show more affective behaviour towards these patients [33,34].

In the observational study from Siminoff et al. (supra), the emotional expressions by physicians varied by patient's demographic variables, with more emotional utterances from their physician [42].

#### Non-verbal behaviour

Non-verbal behaviour is one of the least investigated topics of doctor-patient communication, especially when looking at its interaction with determinants of social class. The effect of non-verbal behaviour is only mentioned in two of the selected articles [41,43]. Non-verbal behaviour can be operationalised in different ways such as eye contact, tone of voice, laughter, facial expression, physical distance, nodding, etc. [26].

The meta-analysis by Hall et al. (supra) could not find any research that was done on the association between the physicians' non-verbal behaviour and the patients' social class [32]. The same year of the Hall review, Street and Buller examined the non-verbal behaviour in doctor-patient interactions and the relationship with patient's age, sex and social class measured as educational level. No differences were found in the level of non-verbal communication towards patients with different educational level. However, when talking to higher educated patients the physicians reciprocated their body orientations more than they did with lower educated patients. Finally, this article refers to specific difficulties in coding non-verbal behaviour, which is much more complex than categorising the verbal interactions [44].

#### Patient-centeredness

Patient-centeredness can be classified into several aspects such as supportive talk, being attentive to patients' psychosocial as well as physical needs, enabling the disclosure of patients' concerns, conveying a sense of partnership and actively facilitating patient involvement in the decision-making [45]. In 10 of the 20 selected articles, patient centeredness in relationship with the Social class of the patient is described.

First of all there is the relationship between patients' social class and the decision making style of the doctor, described in three studies. In a study by Kaplan et al. social class was measured as educational achievement. Patients with a high school education or less were less involved in treatment decisions, less given a sense of control over treatment decisions and less asked to take responsibility for care than patients with post-graduate college education [46]. Also McKinstry observed the patients' preference for shared decision making. Social class was determined by education. Patients' preference for shared decision style or directive approach was associated with their social class, age, the scenario and their perception of the consultation style of their own physician being shared or directive. A lower social class predicted a lower preference for shared decision making style [47]. Murray et al. attempted to determine the congruence between patients' preferred style of clinical decision-making and the style they usually experienced. Social class includes household income and educational achievement. People of high SES were more likely to prefer shared decision-making, and people of low SES were more likely to prefer consumerism and paternalism. Wealthier patients also were more likely to experience their preferred style of shared decision making. The results also point out that SES was strongly associated with reporting having enough information. Respondents who had not completed high school were less likely than those with an advanced degree to report having enough information to make the right decision [48].

Roter et al. Studied the preferred communication style of the patient. They described five communication patterns and their relationship with several patient characteristics, among social class measured as income. Patients approached in the narrowly biomedical pattern were more likely to be poorer than patients approached in other patterns [49]. Jensen et al. surveyed whether literacy, numeracy and optimism are related to satisfaction with health care providers' communication skills. Participants' social class was measured as educational level and admitted as a predictor variable. Almost half of the low-income patients were displeased with the amount of time health providers spent with them during interactions. As displayed in earlier studies, communication dissatisfaction appears to be more common in low-income adults than in higher income adults [50].

Certain aspects of communication can vary widely among different doctors or among patients. Bao et al. aimed to determine the extent to which socio-economic differences (income and education) in cancer screening discussion between a patient and his or her primary care physician are due to inter-physician versus intra-physician variation. Patients with low SES were less likely than their high-SES counterparts to have discussed cancer screening with their physicians. Differences by income are mainly 'between-physicians' While the 'within-physician' differences by income were minimal. The education gradient in cancer screening discussion mainly existed in 'within physicians'. Except for mammogram the rate of discussion more than doubled among college graduates compared with those with a less than high school education. This may indicate that education plays an important role in determining what happens during clinical encounters [51].

Not only the communication style of the physician, but also patient participation in an essential topic in patient-centeredness. Street et al. examined the extent to which patient participation in medical interactions is influenced by the patient's personal characteristics (among social class measured by education), the physician's communication style and the clinical setting. Patients with at least some college education tended to be more active communicators than were less educated patients. Although the more educated persons asked more questions and are more assertive than less educated patients, they do not more often express concerns. The degree to which patients actively participate in medical encounters is a function of multiple patient, physician and contextual factors. It seems that patients are more active participants when interacting with physicians who more frequently engaged in partnership-building and supportive talk [52].

A final aspect described in three studies is building a relationship between patient and physician. The study of Siminoff (supra) et al. shows educational level as significant independent factor on relationship building. It seems that both patients and their physicians spent more time trying to establish an interpersonal relationship with each other. Nevertheless, patients did more effort in relationship building than did their physicians. These results confirm previous evidence that providers communicate differently with patients by education and income [53]. In the study of Maly et al. (supra) on patients with breast cancer both the physician information-giving and patient empowerment in interacting with physicians were found to be significant determinants of breast reconstructive surgery, controlling for possible confounders. These two communication factors diminished the negative influence of education barriers and acculturation [38]. The study of Stewart, where educational level was a measure for social class, showed that physicians were more likely to appeal to the intellect of a patient with a university degree by justifying the drug prescription while on the other hand they offer more emotional support and solidarity to patients with a lower educational level [54]. As presented above, the amount of information given to patients is related to patients' characteristics and to the patients' communicative style. Hereby, the patients' communicative style is not only influenced by his/her educational level but also by the level of partnership building of the physician [33,34]. In the observational study mentioned above by Street et al. it appeared that higher educated patients received more partnership building utterances [33].

## Discussion and conclusion

The aim of the current review is to give the state of the art on the social gradient in doctor-patient communication, to describe which aspects of the consultation are affected by this social gradient, and whether an evolution over time can be noticed comparing the results of older studies with those of newer studies. In this review we found that patients from lower social classes (measured by income, education or occupation) receive less socio-emotional talk, a more directive and a less participatory consulting style characterised by for example less involvement in treatment decisions; a higher percentage of biomedical talk and physicians' question asking; lower patient control over communication; less diagnostic and treatment information and more physical examination. Doctors give more information, more explanations, more (emotional) support and adapt more often a shared decision making style with higher SES participants.

This review also indicates that the literature on the social gradient in doctor-patient communication that was published after 2002, at least addresses new issues and themes. Firstly, in the period 1965-2002, 42 articles were selected for this review, while for the period 2002-2011, 87 articles were selected. These numbers indicate that doctor-patient communication becomes a more emerging topic in the research on delivering qualitative care. Secondly, most of the more recent studies emphasize the importance of the reciprocity of communication: the doctor might communicate differently according to the social status of the patient, and patients may adapt a different communication style according their social class. Patients with a high SES tend to ask more questions, ask for explanations, are more expressive and have a higher level of being opinionated than their lower SES counterparts [33,34,42,55].

Furthermore, there seems to be a growing interest in patient's perception of doctor-patient communication. While in the past, patient's perception was not taking into account or no differences in perception were found, more recent studies show that low SES patients have the feeling doctors fail to explain things in a way they can understand and spend less time with them [21,24,41].

These findings emphasise that doctor-patient communication is a complex interactional system. To depict this complexity, Street et al. (2007) applied an ecological model that takes into account the interplay of multiple physician, patient and contextual factors that collectively influence doctor-patient interactions [56]. The influence of any variable (e.g. ethnicity) may vary depending on the presence of other factors (e.g., the patients' level of education, income, doctors' communication style) [53]. The ecological approach recognizes that within the context of any medical encounter, a number of processes affect the way physicians and patients communicate and perceive one another. There are four important sources of potential influence: the physician's communication style, patients' characteristics, physician-patient demographic concordance and the patients' communication. First, how a physician communicates with a patient may depend on his or her style. Some physicians provide more information, ask more questions, are more supportive and use more partnership-building than other physicians [33,34,49]. Second, variability in physicians' communication and perceptions may be related to the patients' demographic characteristics (education, income, occupation) [57]. Finally, the patients' communication style can have a strong effect on physician behaviour and beliefs [18].

Important in this model is that patient interaction not only depends on the physician's behaviour but also on patients' characteristics and preferences. Patients from lower social classes more often suffer from (multiple) chronic conditions and more severe acute conditions [58]. But also they often have lower levels of health literacy-the degree to which persons have the capacity to obtain, process and understand basic health information and services needed to make appropriate health decisions [59,60]. Furthermore lower social class is associated with a lower sense of personal control also known as external locus of control. This means that the person perceives that certain events such as health and sickness are beyond his/her control [61]. This might explain why low SES people show lower levels of participation. Also, because they are less used to or feel less capable to interact during consultation, they might prefer a more directive consultation style. Recently, an international consortium of research teams in the UK, the Netherlands, Italy and Belgium set up the Gulliver study which focuses on the patient's preferences in doctor-patient communication. Analysing a possible social gradient in these preferences is hereby one of the points of attention of the researchers.

### Limitations of the study

Many of the limitations the review of 2005 encountered are still applicable today. Affective and non-verbal behaviour are important aspects in physician-patient communication e.g. through their influence on patient satisfaction [44]. Still a limited number of studies described the interaction between social class and non-verbal physician-patient communication. All studies indicates the difficulties measuring and coding non-verbal behaviour. Therefore, these limited number of studies entails important methodological difficulties and does not allow us to draw conclusions concerning non-verbal and affective behaviour. Further research on this topic is still needed.

Secondly, it is very difficult to compare the results of the studies due to the great diversity of measurements and frameworks organising these measurements in the different studies. Socio-economic status of the patient was measured by means of educational level, income or occupation [3,62]. An alternative to determine SES is to use "proxy" measures e.g. the insurance status, house tenure, car ownership, socio-demographic measures (race, etc.). Articles using proxy-variables as the only measure for SES were excluded. However, some of the selected articles used these variables in combination with educational level, income or occupational class. Next to the SES of the patient, also communication variables can be classified in many different ways. The variables used in these classifications are not always comparable, making if very difficult to compare the studies using different classification systems. We chose to categorise most of the communication variables according to the axis verbal/non-verbal behaviour. The determinants of communication that did not fit into the categories of this axis were related to patient centeredness.

In order to improve the comparability of future research, the use of a uniform definition and classification of communication variables is indispensable.

## Practice implications

This review of the literature has revealed the complex relationship of doctor-patient communication and reinforces the practice implications of the former review. Physicians behave differently with patients from different SES and patients communicate differently with their doctor depending on their SES. The finding that the physician's communicative behaviour is related to the communicative style of the patient and to his/her personal or social characteristics, may have important implications for the daily practice of the physician.

Physicians need to be aware of the differences in giving information to and involving patients from lower social classes in the consultation, as well as of the underlying causes [63]. It is important that physicians pay attention to the attitudes that they have toward patients, and have to remain aware of how their feelings might impact their behaviour and thus be perceived by patients [64]. They should consider the possibility that conscious or unconscious stereotyping may influence their behaviours, including their interpersonal style [65]. Physicians have to encourage patients to discuss their concerns and to ask questions, and they should listen actively. Communication skills and attitudes training can be an important tool to improve these defaults: the effects of such training have been proven and can persist over time [66].

Patients have a certain power to control communication during the consultation and to influence the physicians' communicative behaviour. However, patients from lower social classes seem to exercise this control less than patients from higher educated groups. It seems that not only patient's personal characteristics but more importantly their communicative behaviour has an influence on the doctor-patient communication. Therefore it is important to empower the patients towards more self-efficacy and towards learning how to express their concerns and preferences [33,34,38,67].

It has been shown that interventions to increase the participation of patients with low education obtain a good response and lead to measurable and clinically important improvements in health outcomes [68]. By understanding processes that facilitate or hinder patient involvement, physicians should be better able to adapt their own communication and office practices to help patients more effectively participate in medical encounters [52].

On the other hand, it is important not to take patient-centered care as the obvious and only choice. When working patient-centered, physicians should not only focus on normative thinking regarding participatory decision making but they also have to pay greater attention to a broader set of considerations relating to respect for patients as individuals. It is important to enable and empower patients but it is perhaps even more important to enable and empower them to the degree that they desire. As McKinstry states: "*Doctors need both communication skills and time in consultations, along with knowledge of their patients, to determine at which times, with which illnesses, and at which level their patients wish to be involved in decision making" *[47].

Future research should further investigate low-SES patients' perceptions and expectations of their health care providers' communication skills and of being involved in the decision making process. Special attention should be paid to the relationship between patient skills, patient activism and communication satisfaction [50].

## Competing interests

The authors declare that they have no competing interests.

## Authors' contributions and acknowledgements

VE, NdeL, WS and MdeS equally contributed to the research and the report of the study, DM helped formulating the core idea and was involved in the final editing of the manuscript. All authors read and approved the final manuscript
